# Getting It Right in Obstructive Lung Disease

**DOI:** 10.3390/jcm12083032

**Published:** 2023-04-21

**Authors:** Annalisa Carlucci, Barbara Fusar Poli

**Affiliations:** 1 Dipartimento di Medicina e Chirurgia, Università Insubria, 21100 Varese, Italy; 2 Pneumologia Riabilitativa Istituti Clinici Scientifici Maugeri, 27100 Pavia, Italy

**Keywords:** chronic obstructive pulmonary disease, hypoventilation, non-invasive ventilation, chronic hypercapnic respiratory failure

## Abstract

Chronic hypercapnic respiratory failure in obstructive lung diseases may benefit from nocturnal Home non-invasive ventilation (HNIV). It has been shown that in patients with persistence of hypercapnia after an acute episode of chronic obstructive pulmonary disease (COPD) exacerbation requiring mechanical ventilation, HNIV may improve the risk for new admission and survival. The ability to reach these aims depends on the correct timing of enrolling patients, as well as a correct definition of ventilatory needing and setting of the ventilator. This review tries to define a possible home treatment path of hypercapnic respiratory failure in COPD by analyzing the main studies published in recent years.

## 1. Introduction

Chronic hypercapnic respiratory failure in lung diseases usually represents a reason to consider the prescription of home noninvasive ventilation (HNIV). When to start it and how to get it depends on the underlying chronic respiratory disease and the available clinical evidence. The aim of this review is to focus on indications and modes of using NIV at home in patients with chronic respiratory failure secondary to obstructive lung disease (in this review, we will mainly consider COPD patients). We will start with a clinical case to follow a possible “journey” of a patient with chronic respiratory failure who is a possible candidate for home ventilation.

## 2. A Clinical Journey of a Typical Patient

A 68-year-old patient was hospitalized for an acute exacerbation of COPD (AECOPD) with acute respiratory failure and acidosis. During an initial observation in the emergency room, after stabilization with oxygen therapy targeted to keep an oxygen saturation between 89 and 92%, as indicated by BTS guidelines [1], arterial blood gases (ABGs) with a FiO_2_ = 0.31 showed a PaCO_2_ of 85 mmHg, PaO_2_ of 65 mmHg and pH of 7.21. Lung ultrasound examination, targeted to detect specific ultrasound patterns according to international recommendations [2], excluded pulmonary edema, pneumonia and pleural effusion, while blood tests showed a significant increase of the C-reactive protein (CRP) and neutrophilic leukocytosis. For this reason, antibiotic therapy and oral corticosteroids were started, together with non-invasive ventilation (NIV), in line with international guidelines [3]. In the next five days of hospitalization, the patient progressively improved, as did their laboratory tests and ABGs. However, two days after weaning from NIV, the PaCO_2_ was still elevated (56 mmHg), despite a normalization of the pH (7.37). For this reason, physicians considered the opportunity to prescribe NIV at home to facilitate the discharge of the patient.

## 3. Methods: What the Literature Tells Us

We conducted a comprehensive PubMed search of full-length articles in English using the following key terms: “chronic hypercapnic respiratory failure” or “COPD” and “home non-invasive ventilation”. We considered RCTs and physiological studies published since 2000, as well as European and American recommendations on the application of HNIV in chronic respiratory failure for patients with hypercapnic COPD. We excluded articles published in non-peer-reviewed journals. Papers were independently reviewed by the two authors, and those judged to be more informative for the purpose of the review were retained.

## 4. Phenotype-Based Indications to HNIV

### 4.1. Stable Hypercapnic Respiratory Failure after Severe Exacerbation

Patients with AECOPD needing mechanical ventilation who survive after hospitalization have a high risk of readmission and death. More than sixty percent of them may have another life-threatening event, and 50%, on average, may die mainly due to respiratory failure [4]. For this reason, in 2010, a pilot study considering this subgroup of 57 patients randomized them to be treated at home with HNIV or sham ventilation (continuous positive airway pressure (CPAP) at 4 cmH_2_O). This promising study showed a significant reduction of new readmission for AECOPD in the group treated with NIV (38.5% in the NIV group vs. 60.2% in the control group, *p* = 0.039). However, there was an unclear definition of AECOPD, and the very small sample size and high drop-out rate (25% of enrolled patients) made the results difficult to apply in clinical practice. Moreover, NIV or control therapy was initiated before hospital discharge after more than 48 h of successful weaning. Some years later, two large-scale international randomized control trials (RCTs) investigated the efficacy of HNIV in reducing mortality and readmission rates after an AECOPD compared to standard treatment [5,6]. In both studies, setting NIV was targeted to achieve normocapnia [5] or to achieve control of nocturnal hypoventilation with a high-pressure ventilation strategy [6]. The RESCUE trial, involving 201 hypercapnic COPD patients, failed to detect any differences in the main outcomes (exacerbations and deaths) between the two groups during the 1-year follow-up [5]. Interestingly, both groups showed a significant reduction of PCO_2_ after 3 months. In contrast, the HOT-HMV trial [6] found a remarkable difference in the 1-year risk of readmission or death combined outcome (63.4% in the NIV group vs. 80.4% in the standard therapy group) and in the median time to readmission or death (4.3 months vs. 1.4 months, respectively). Possible explanations of this huge discrepancy between the two studies may be the different definition of hypercapnia (>45 mmHg in RESCUE vs. >53 mmHg in the HOT-HMV trial), as well as different timepoints for the initial assessment and enrollment of patients. In the RESCUE trial, randomization was carried out 48 h after weaning from the acute treatment of NIV, while in the HOT-HMV trial, the assessment of hypercapnia for inclusion in the trial was carried out 2–4 weeks after discharge. These latter criteria were to rule out patients who spontaneously reverted their PaCO_2_ within a normal range in the weeks following their acute exacerbation. The normalizing of PaCO_2_ post-exacerbation was already described by an observational study many years ago [7] and that perhaps masked the beneficial effects of NIV in the right population in the RESCUE study. Some years before, Funk et al. used a reverse approach; they provided HNIV therapy to 26 consecutive patients who remained hypercapnic after an episode of AECOPD, and after 6 months of therapy, they randomized the participants to either continue or withdraw from HNIV [8]. They showed a higher risk of clinical worsening in the withdrawal group and a more significant reduction in the walking distance compared to the HNIV group.

To standardize indications to HNIV, a recent European Task Force suggested, with a conditional recommendation, that HNIV should be used after an AECOPD episode requiring acute NIV if hypercapnia persists in the follow-up after 2–4 weeks [9]. However, the panel did not consider the possible overlap with obstructive sleep apnea (OSA). It has been shown, in fact, that OSA may be a possible cause of persistent hypercapnia but also of the severity of AECOPD leading to mechanical ventilation [10,11]. Some years ago, Marin and co-workers showed that patients with overlap syndrome had a higher risk of hospitalization and death compared to COPD with the same severity of respiratory dysfunction and the same inhaler therapy. They also showed that in these patients, treatment with CPAP can reduce this risk, reporting it at the rate related to underlying lung disease [12]. Moreover, it is already known that in hypercapnic overlap patients, home CPAP may be able to revert hypercapnia in a few days of treatment and maintain a normal PaCO_2_ value for at least the first year of treatment [13].

Both previously mentioned RCTs, RESCUE and HOT-HMV, excluded patients with OSA. However, the criteria to define OSA were different and did not consider a standardized use of polysomnography as the gold standard. In fact, in the RESCUE study, overnight polygraphy or polysomnography was suggested in patients with a body mass index ≥30 kg/m^2^, or in patients with complaints of excessive snoring, disrupted sleep or morning headache. The HOT-HMV trials left individual centers to use established pathways for OSA screening: patients in which there was a clinical suspicion of OSA based on clinical review or overnight oximetry underwent further testing with limited respiratory polygraphy. However, it has been shown that the use of a specific screening instrument, such as sleepiness scales, would miss nearly half of patients with severe OSA [14]. Similarly, in a cohort of COPD patients coming from an AECOPD, more than 40% of those having moderate to severe OSA at a polygraphy screening had a BMI <30 kg/m^2^ [10]. This raised the doubt that OSA was not correctly screened and overlap patients may be included.

**Figure 1:**
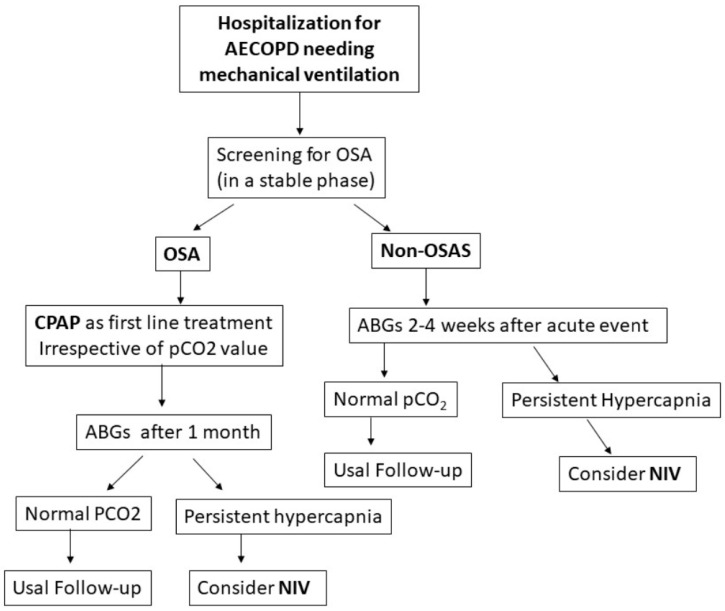
Shows a suggested algorithm for the management of patients after acute exacerbation of chronic obstructive pulmonary disease (AECOPD). Once patients are clinically stable (improved symptoms, normal pH despite weaning from NIV, no more need for antibiotics or oral steroids, no signs of cardiovascular decompensation), they should be screened for obstructive sleep apnea (OSA). Those diagnosed with OSA should start with continuous positive airway pressure (CPAP) therapy. Then, be followed up with as per the algorithm. Non-OSA patients should have arterial blood gases (ABG’s) one month post-exacerbation and be managed as per the flow chart. NIV is non-invasive ventilation.

For this reason, the most recent American Thoracic Society guideline on HNIV in chronic hypercapnic COPD patients [14] highlighted the importance of screening the patient for OSA before starting HNIV. A proposed plan to follow and define the need for HNIV in this subgroup of patients is reported in Figure 1.

### 4.2. Stable Hypercapnic Respiratory Failure

Another phenotype of chronic hypercapnic respiratory failure is represented by COPD patients who have not experienced hospitalization due to severe acute exacerbation. Several studies have investigated the effects of long-term HNIV on various outcomes in hypercapnic stable COPD, including mortality, hospitalization rate, and patient-centered outcomes, such as exercise tolerance, symptom severity or health-related quality of life (HRQL) [9,14]. However, differences in study, selection criteria (such as level of hypercapnia), patient population (inclusion or not of obese patients or screening for OSA), and above all setting of NIV (aimed or not to significantly reduce or normalize PaCO_2_) are the main reasons for the controversial results. The first RCTs [15,16] enrolled very small groups of patients with mild to moderate hypercapnia. The sample size was not tailored based on the primary outcome that was not really defined. In both studies, the authors did not find a survival benefit when HNV was added to standard COPD care. Some years later, McEvoy [17] found, in a cohort of 144 patients, no positive survival effect by additional HNIV after 2 years in the intention-to-treat analysis. However, in the per-protocol analysis (i.e., considering patients who used NIV for more than 4 h/night), the survival advantage was slightly greater, suggesting that the nightly duration of NIV treatment was an important determinant of outcome. This happened despite a slightly worsened quality of life and no significant improvement in pCO_2_ value. These RCTs defined a phase of clinical research in which not much emphasis was placed on the setting of NIV.

The more recent multicenter RCT included 195 patients with stable chronic hypercapnia (PCO_2_ > 51.9 mmHg) and randomized them to either HNIV or standard therapy [18]. The HNIV setting, particularly the Inspiratory Positive Airway Pressure (IPAP), used was targeted to obtain a PaCO_2_ reduction of at least 20% from baseline or to achieve PaCO_2_ <48 mmHg. The trial was terminated before the target sample size of 300 patients was attained due to a change in the national guidelines for NIV provision supporting the use of HNIV. One hundred and ninety-five patients were enrolled in almost 5 years. The results showed a 1-year survival benefit in patients randomized to HNIV with an increase in HRQL. Surprisingly, the standard therapy group showed a three-month mortality rate of around 30% despite having a relatively well-preserved exercise capacity (6-min walking distance over 200 m) and low emergency admission rates. The main differences in the enrolled criteria and outcomes among these studies are shown in Table 1.

Pooled data analysis coming from RCTs included in the two task forces led the panel groups to suggest treating these patients as a conditional recommendation.

In fact, even though the results regarding mortality and hospitalization rate from the 13 RCT studies were inconclusive, the improvement of exercise tolerance and quality of life and the reduction of dyspnea seem to be stronger and were considered paramount effects of HNIV in this subgroup of patients. Even less evidence is present regarding the level of pCO2 as a cut-off for the definition of hypercapnia for which there is no consensus.

## 5. Returning to Our Patient’s Journey

Due to the persistence of hypercapnia after NIV weaning, the patient was discharged with HNIV. The setting chosen was spontaneous/timed mode with an inspiratory positive airway pressure (IPAP) of 25 cmH_2_O and expiratory positive airway pressure (EPAP) of 5 cmH_2_O, back-up rate = 12 breaths/minute. He was discharged to a rehabilitation center due to the significant reduction of exercise tolerance and compromised activities of daily living. The attending physician decided to stop nocturnal NIV, wait and follow up with ABG while pulmonary rehabilitation was carried out. After 2 weeks, the ABGs showed a normal value of PaCO_2_ (42 mmHg) with a normal pH (7.40). Polysomnography was used to diagnose a severe OSA with an apnea/hypopnea index of 56 events/minute. The patient underwent a CPAP titration study, and the patient was discharged with a CPAP set at 12 cmH_2_O. His follow-up at 6 and 12 months confirmed that he had stable normal PaCO_2_ level on ABGs.

## 6. How to Ventilate: Choosing a Ventilator and Setting for Successful Ventilation

A pressure-targeted mode is the most frequently used mode of ventilation during HNIV in chronic hypercapnic COPD. However, some years ago, several studies failed to show a significant reduction of PaCO_2_ during treatment [15,16,17], raising the issue of inappropriate settings. For this reason, a strategy of titrating inspiratory pressure at very high values and respiratory rate (RR) close to the spontaneous one of the patients was shown to be significantly superior to the usual setting (Low Intensity), not only in reducing PaCO_2_ but also in improving clinical outcomes (dyspnea, forced expiratory volume at 1 s and exercise performance) with an unexpected, better tolerability [19]. The new concept of “high intensity” ventilation was therefore introduced, meaning to use the best combination of highest inspiratory pressure and respiratory rate able to normalize arterial pCO_2_ or improve it at least of 20% of the baseline value. Later, *Murphy et al.* showed that high pressure with a low respiratory rate was able to reach the same results as high intensity [20]. However, to date, no study has compared high intensity to low intensity ventilation in terms of mortality and exacerbation rate as primary outcomes, and no study has compared strategies targeting PaCO_2_ reduction versus those that did not. Hence, we do not know if the clinical and physiological effects of HNIV are all mediated by the PaCO_2_ reduction. On the other hand, one previous study showed a survival benefit without a change in hypercapnia [17]. Moreover, more recently, Theunisse and co-workers showed that low pressure HNIV aimed to optimize acceptance and adherence may significantly reduce admission rate, duration of hospitalization, symptoms and quality of life at 1 year in severe COPD patients irrespective of the presence of hypercapnia [21]. Therefore, further investigations are needed to show if chronic hypercapnia and the reduction or normalization of PaCO_2_ should be considered, respectively, the only enrollment criteria and target of successful HNIV. Concerning the NIV setting, the European Task Force [9] suggests titrating HNIV to normalize or reduce PaCO_2_ levels in patients with COPD as a conditional recommendation with very low certainty evidence. Adherence to HNIV is another crucial point that can determine the success of this treatment. For COPD patients, an increased adherence to the therapy up to 20 h/day was shown to significantly correlate with a lower mortality rate [22].

## 7. Conclusions

In chronic hypercapnic COPD, the indication for HNIV is based on very low certainty. It is strongly recommended that polygraphy be carried out to exclude OSA in patients who were hospitalized for an AECOPD requiring mechanical ventilation. Inspiratory pressures high enough to significantly improve hypercapnia or to restore normocapnia should be accurately titrated. Having clear objectives of HNIV more than PaCO_2_ reduction (quality of life, dyspnea and exercise tolerance, exacerbation rate) is mandatory to increase the cost–benefit ratio of HNIV. Adherence to the therapy may be a crucial point in reaching all established objectives.

## Figures and Tables

**Table 1:** Shows a summary of the characteristics and main results of the RCTs about the effect of HNIV on stable hypercapnic respiratory failure.

	Number of Patients	Basal PCO_2_	Final PCO_2_	IPAP/EPAP (cmH_2_O)	1-Year Death	Hospital Admission	Exacerbation	QoL	QoS	Dyspnea	Exercise Tolerance
Casanova [15]	44	50.7	51.1	12/4	unchanged	Reduced at 3 months Unchanged at 1 year	unchanged	NA	NA	Improved at 3 and 6 months, unchanged at 1 year	NA
Clini [16]	90	54	52	14/2	unchanged	unchanged	NA	improved	unchanged	Improved	unchanged
McEvoy [17]	144	54.1	53.2	12.9/5.1	Unchanged; Improved in pts using NIV > 4 h/day	NA	NA	worsened	NA	VN	NA
Kohnlein [18]	195	58.4	48.7	21.6/4.8	improved	unchanged	NA	improved	NA	NA	unchanged

IPAP = inspiratory positive airway pressure; EPAP = expiratory positive airway pressure; LTOT: long-term oxygen therapy; QoL = quality of life; QoS = quality of sleep; NA = not applicable.

## Data Availability

No new data were created or analyzed in this study. Data sharing is not applicable to this article.
